# The gut–brain axis in pharmacology: microbiome-driven modulation of CNS drugs and neuropsychiatric outcomes

**DOI:** 10.3389/fphar.2026.1826681

**Published:** 2026-05-12

**Authors:** Tao Xu, Samaresh Pal Roy, Rajesh Hadia, Annu, Suchismita Bhowmik, Ayush Panchal

**Affiliations:** 1 Hunan Provincial Key Laboratory of the Traditional Chinese Medicine Agricultural Biogenomics, Changsha Medical University, Changsha, China; 2 Department of Pharmacology, Sardar Patel College of Pharmacy, Anand, Gujarat, India; 3 Department of Pharmacy Practice, Sumandeep Vidyapeeth Deemed to be University, Vadodara, Gujarat, India; 4 Department of Pharmaceutics, Sardar Patel College of Pharmacy, Anand, Gujarat, India; 5 Institute of Nursing, Charotar University of Science and Technology, Changa, Gujarat, India; 6 School of Life and Medical Sciences, University of Hertfordshire, Hatfield, United Kingdom

**Keywords:** antidepressants, CNS pharmacology, gut–brain axis, microbiome, psychobiotics

## Abstract

**Background:**

The gut–brain axis has emerged as a critical regulator of Central Nervous System (CNS) pharmacology, significantly influencing drug response and neuropsychiatric outcomes through complex interactions between the gut microbiota and host systems.

**Objective:**

This review aims to examine the role of gut microbiota in modulating the pharmacokinetics and pharmacodynamics of CNS drugs, and to explore their implications in neuropsychiatric disorders.

**Key findings:**

Accumulating evidence indicates that microbial enzymes and metabolites can alter drug absorption, metabolism, and bioavailability, particularly for antidepressants such as Selective Serotonin Reuptake Inhibitors (SSRIs) and Tricyclic Antidepressants (TCAs). In turn, psychotropic medications can modify gut microbial composition, leading to dysbiosis and variability in therapeutic outcomes. Microbiota-derived metabolites, immune signaling pathways, and host genetic factors (e.g., cytochrome P450 polymorphisms) collectively contribute to interindividual differences in drug efficacy and safety.

**Conclusion:**

Integration of microbiome profiling into pharmacological research holds significant potential for advancing precision medicine in neuropsychiatry. However, challenges such as methodological heterogeneity, limited longitudinal clinical data, and lack of standardized biomarkers must be addressed to enable clinical translation.

## 1 Introduction

The gut-brain axis, which connects the gastrointestinal tract to the central nervous system, regulates neurological, metabolic, and behavioral functions. This axis’s complex neuronal, endocrine, immunological, and microbial pathways allow the CNS to modulate gut motility, secretion, and microbial ecology and gut microbiota-produced inflammatory signals and metabolites to affect brain physiology (Młynarska et al., 2022; Góralczyk-Bińkowska et al., 2022; Marazziti et al., 2021). found that gut microbiota composition and function influence interactions and are linked to major psychiatric disorders such as anxiety, depression, ASD, and cognitive dysfunction. Gut microbiota metabolites such as glutamate, indoles, tryptophan derivatives, and short-chain fatty acids affect neurotransmission, neuroinflammation, and synaptic plasticity (Suganya and Koo, 2020; Ahmed et al., 2022).

Despite its neurological importance, the gut-brain axis’s effects on modern pharmaceuticals have been little studied. There is growing evidence that the gut microbiome may alter how CNS drugs are absorbed, digested, changed, and treated. Modern mental health research agrees that microbiome makeup may explain psychiatric medication treatment response heterogeneity (Lee et al., 2022; Kargbo, 2023). Major depressive illness microbial fingerprints predict therapy response and side effects (Jiang et al., 2024; Sjöstedt et al., 2021).

SSRI efficacy may vary due to microbial regulation of serotonergic pathway and psychiatric drugs also change gut microbiota. A symbiotic relationship might increase side effects or diminish long-term therapy efficacy due to drug-induced dysbiosis (Zhang et al., 2021; Ramsteijn et al., 2020). found that chronic amitriptyline and fluoxetine exposure may alter gut microbial diversity, metabolic pathways, and inflammatory markers, causing tolerance, tachyphylaxis, and gastrointestinal issues. According to this new pharmaco-microbiology theory, knowing how drugs act in the CNS requires considering the microbiota. Microbiome research is growing, yet many concerns remain. Most research has focused on developmental, neurodegenerative, or psychiatric pathways, not drug-microbiome interactions or microbial regulation on CNS drug bioavailability and neuropsychiatric effects. With personalized or precision medicine, microbial signatures must be considered when making therapeutic decisions (Yaqub et al., 2025). This review summarizes the latest studies on microbiome-driven modulation of central nervous system pharmacology, including how gut microbiota affect drug efficacy, neuropsychiatric consequences, and new treatment methods. Pharmacologically, this work indicates how microbiota-guided therapy can change tailored mental healthcare and CNS drug development.

### 1.1 Novelty and contribution of this review

In contrast to prior reviews that have primarily addressed general mechanisms at the gut-brain axis, this review specifically integrates microbiome-mediated modulation of CNS pharmacokinetics and pharmacodynamics with neuropsychiatric outcomes (Kargbo, 2023; Lee et al., 2022). It focuses on bidirectional drug-microbiome interactions, offers more details on pharmacomicrobiomics in antidepressant variability and explores a translational approach connecting microbial metabolites to clinical pharmacology. In addition, the review also highlights novel approaches, including microbiome-based precision medicine and nanomedicine-based drug delivery systems.

### 1.2 Methodology for literature selection

To compile existing evidence on microbiome-drug interaction in CNS pharmacology, this review was done via a structured narrative review method. The databases, such as PubMed, Scopus, and Web of Science, were used to find the relevant literature on the topic written in 2015 and 2025.

The keywords were gut-brain axis, microbiome, CNS drugs, SSRI microbiome interaction, psychobiotics, and neuroinflammation microbiota.

- Criteria of included studies included,
- Peer-reviewed original research articles and reviews.
- Studies evaluating microbiome–drug interactions or neuropsychiatric outcomes were included.
- Preclinical and clinical research with CNS-active drugs.

The exclusion criteria were.

- Non-English publications.
- The studies that were not mechanistic or clinically relevant.

One hundred and twenty studies were incorporated following the screening of titles, abstracts, and the full texts. These findings were thematically arranged into the pharmacokinetics, pharmacodynamics, disease implications and therapeutic interventions.

## 2 The gut–brain axis: biological architecture and communication pathways

### 2.1 Neural, endocrine, and immune pathways

The vagus nerve, one of numerous gut-brain sensory routes, sends messages from the digestive tract to the brain. Młynarska et al.(2022) , Góralczyk-Bińkowska et al.(2022) found that vagal afferents affect mood, stress response, and cognition when exposed to inflammatory signals and microbial metabolites. Stress affects the endocrine system and hypothalamic-pituitary-adrenal [HPA] axis. In mental illnesses, dysbiosis hyperactivates the HPA axis, causing behavioral abnormalities and increased cortisol release (Marazziti et al., 2021; Ahmed et al., 2022).

This figure highlights major pathways along the gut-brain axis, including neural (vagus nerve signaling), immune (cytokine-mediated inflammation), endocrine (activation of the Hypothalamic-Pituitary-Adrenal [HPA]), and metabolic (microbially produced metabolites such as short-chain fatty acids) mechanisms by which the gut microbiota communicate with the central nervous system.

The gut microbiota and the central nervous system, which is mediated by the gut-brain axis, contribute to neuroinflammatory events. Microbial changes may trigger peripheral immune mechanisms, enhance production of cytokines, and impair the integrity of the blood-brain barrier, eventually facilitating neuroinflammation. Neuroinflammatory cascades connected to neurodegenerative disorders, anxiety, and depression are triggered by microbiome changes that excite the peripheral immune system, boost cytokine production, and weaken the blood-brain barrier (Bicknell et al., 2023; Niccolai et al., 2019). New research suggests that immune system-gut microbiota interactions can affect microglial activation and neurocognitive performance over time. These pathways demonstrate how the microbiota affects immunological, hormonal, and neurological system function.

**FIGURE 1:**
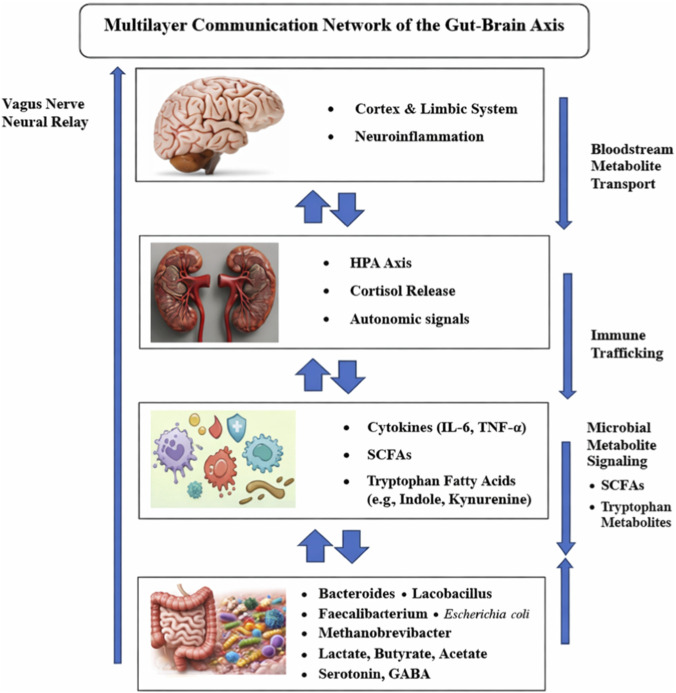
The gut–brain axis communication pathways.

Figure 1 The gut -brain axis represents a layer 1 point-to-point communication network between the gastrointestinal tract and the central nervous system. The drawing depicts the presence of some of the most important organs such as the brain and the gut, which are connected by means of some neural pathways such as the vagus nerve, endocrine signaling through the hypothalamic-pituitary-adrenal (HPA) axis and immune mechanisms involving cytokines. Arrows show bidirectional signaling, and the production of metabolites such as short-chain fatty acids and neurotransmitter precursors that affect brain work. The figure also indicates the integrated contribution of neural, hormonal, and microbial interactions in the regulation of neurophysiology and behavior.

### 2.2 Microbial metabolites as neuroactive molecules

Many gut microbiota neuroactive chemicals and structural mechanisms affect brain function. Butyrate and propionate affect microglial growth, neurotransmitter synthesis, and the blood-brain barrier; their significance in brain stability is highlighted (Gruenbaum et al., 2024; Ahmed et al., 2022). Gut microbiota converts tryptophan into indoles, kynurenine derivatives, and serotonin precursors, affecting mood, cognition, and stress response (Lee et al., 2017; Afifi et al., 2016). Indole-3-propionate protects neurons, while tryptophan metabolism abnormalities cause depression and SSRI (Selective Serotonin Reuptake Inhibitor) insensitivity (Ahmed et al., 2022; Hulme et al., 2020).

The microbiome affects neuroplasticity and excitotoxicity through glutamatergic signaling. Recently, gut microbiota-produced metabolites have been linked to neurotoxicity, cognitive health, and receptor function and glutamate turnover (Gruenbaum et al., 2024). Stomach microorganisms create and release hydrogen sulfide (Petra et al., 2015), which protects and destroys neurons as a gaseous neuromodulator. According to Munteanu et al. (2024), altering H_2_S production can damage mitochondrial integrity and inflammatory signaling, potentially leading to Alzheimer’s and Parkinson’s disease. Metabolite-driven pathways reveal how gut microbiota influences brain cells, synapses, and molecules (Iannone et al., 2019; Warner, 2019).

### 2.3 Early-life microbiome and CNS development

Early brain and behavioral development are affected by gut microbiota (Yarandi et al., 2016). Initial microbial colonization disturbance by delivery route, antibiotics, food, or environment alters synaptic wiring, neuroimmune calibration, and stress response (Cenit et al., 2017). Early life microbial patterns are associated with autism, ADHD, and anxiety (Kerna et al., 2024; Marano et al., 2025). Microbiome-brain interactions during infancy alter neuronal maturation, microglial proliferation, and neurotransmitter programming, preparing the brain for emotional and cognitive development (Zareifopoulos and Panayiotakopoulos, 2017; Petropoulos et al., 2025; Młynarska et al., 2025) found microbial profiles connected to behavioral problems, immune system malfunction, and gastrointestinal disorders in children with autism spectrum disorder, suggesting this association (Jabbari Shiadeh et al., 2025). relate neuropsychiatric vulnerability to maternal microbiome, placental immunological systems, and prenatal and postnatal changes. These findings show that early microbial habitats affect CNS health and neurodevelopment throughout life.

Together, these collective mechanisms associated with the gut-brain axis represent the biological basis through which microbial activity can modulate pharmacokinetics and pharmacodynamics of CNS drugs and ultimately lead to changes in neuropsychiatric outcomes.

## 3 Microbiome–driven modulation of CNS drugs

### 3.1 Pharmacokinetics and pharmacodynamics interactions

Building on these biological mechanisms, the gut microbiome directly influences CNS pharmacology by modulating drug absorption and metabolism as well as their therapeutic effects.

Human medication fate increasingly depends on gut microbiota. Microbial enzymes, transporters, and metabolites significantly influence CNS-active drug PK(Pharmacokinetics) and PD (Pharmacodynamics), according to Doroszkiewicz et al. (2021), Anand et al. (2022). Research shows that gut bacteria degrade, biotransform, and compete for metabolic pathways to metabolize xenobiotics (Scriven et al., 2018; Generoso et al., 2020). This defies classical pharmacology’s host physiology-controlled drug absorption and metabolism (Kargbo, 2023; Lee et al., 2022). Interactions affect treatment results, systemic exposure, and pharmaceutical absorption.

Gut microbiota enzymes directly degrade CNS medications, making them more active, less active, or toxic (Grochowska et al., 2018; Principi and Esposito, 2016). Microbiota changes affect intestinal pH, barrier integrity, and efflux transporter expression, affecting medication absorption (Queiroz et al., 2022; Kavvadia et al., 2017). Due to interindividual treatment response variability, antidepressant pharmacology emphasizes this microbial role.

Drug biotransformation is mainly influenced by microbial enzymes, including β-glucuronidases, azoreductases and dehydroxylases at the molecular level, which directly affect drug bioavailability and systemic exposure (Zhang et al., 2018; Kargbo, 2023). Microbial metabolites such as short-chain fatty acids grant access to intestinal epithelial tight junctions and drug transporter proteins (i.e., P-glycoprotein) that can influence the absorption of drugs (Vernia et al., 2021).

Microbiota-derived metabolites (e.g., butyrate, propionate) and secondary bile acids have been reported to regulate hepatic cytochrome P450 enzymes, especially CYP1A2, CYP2C9, and CYP3A4, by regulating epigenetic processes (e.g., PXR and CAR activation) and nuclear receptor signaling pathways (Lee et al., 2022; Walsh et al., 2018). Such interactions have the potential to change the rate of metabolism and systemic exposure to drugs, thus affecting therapeutic efficacy and toxicity.

Certain bacteria can break down SSRIs like citalopram, sertraline, and fluoxetine, altering their effects (Sherwin et al., 2016; Shobeiri et al., 2022). SSRIs, which target CNS transporters, can be reduced by gut microbiota metabolism before they enter the bloodstream. While SSRIs can selectively enhance or reduce gut bacteria, they are antimicrobials. Symbiotic interactions confound pharmacodynamic predictions (Sajdel-Sulkowska, 2021; Arneth, 2018). The gut microbiota and TCAs like amitriptyline interact in a two-way. In animals and humans, amitriptyline lowers microbial diversity and affects metabolic pathways (Deters and Saleem, 2021). However, microbial metabolism may affect amitriptyline’s pharmacokinetics, including its conversion to nortriptyline. The microbiota affects CNS medication response by enzymatic breakdown, immunological route regulation, and neuroactive metabolite production, complex drug-specific interface (Quagliariello et al., 2018; Woo et al., 2020).

### 3.2 Critical perspective on conflicting evidence

Despite the fact that some research reveals that gut microbiota increases the metabolism and efficacy of drugs, others show decreased bioavailability as the result of microbial degradation (Zhang et al., 2018; Kargbo, 2023). Such conflicting results can probably be explained by the interindividual differences in the microbial composition, disparity in the study design and by the drug-specific metabolic pathway.

Particularly, the comparative role of gut microbiota as compared to host genetic determinants, especially cytochrome P450 (CYP) polymorphisms has not been adequately investigated. Although direct biotransformation of a drug can be done by microbial enzymes, the host genetics might dominate the systemic metabolism (Grochowska et al., 2018; Principi and Esposito, 2016). There is need to study in the future to combine microbiome profiling with pharmacogenomics to unravel these interactions.

CNS drugs, as well as the interaction between CNS drugs and the microbiome, are a two-way process. Although gut microbiota is capable of metabolism and drug activity modification, pharmacological agents are capable of changing microbial diversity and activity (Sjöstedt et al., 2021). This two-way interaction produces a dynamic feedback loop that determines the effectiveness of therapeutic and undesirable outcomes, which is why pharmacomicrobiomic models should be integrated.

### 3.3 Microbiome influence on antidepressant efficacy

**FIGURE 2:**
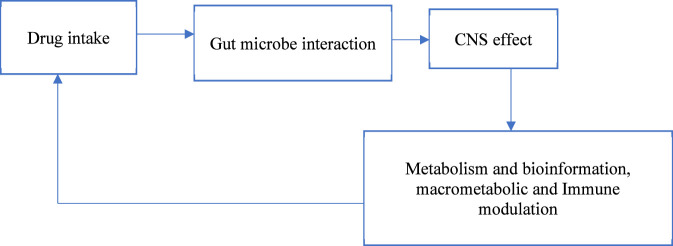
Schematic representation of microbiome–drug interaction pathways illustrating drug metabolism, microbial modulation, and feedback effects on CNS pharmacology.

Clinical trials and fundamental research reveal that the gut flora affects antidepressant efficacy (Masanetz et al., 2022; Zhu et al., 2017; Honarpisheh et al., 2022). The baseline microbiome can predict SSRI response, with some taxa of bacteria being related to better or worse outcomes. This link appears to be driven by microbial modulation of serotonin metabolism, inflammatory signaling, and neuroplasticity, which are crucial to SSRI activity. According to Jiang et al. (2024), Walsh et al. (2018), microbial metabolites affect serotonergic neurotransmission and tryptophan metabolism, affecting antidepressant efficacy in major depressive disorder. Figure 2 illustrating how the intake of drugs reacts with gut microbiota resulting in microbial metabolism that influences neurotransmission, immune responses, metabolism and bioavailability. The bidirectional interaction between drug response and microbial composition can be emphasized by a feedback loop. Microbial profiles with decreased proinflammatory taxa or improved tryptophan-to-indole pathways improve treatment effects (Abautret-Daly et al., 2018; Clapp et al., 2017). Thus, microbiota-derived indicators are emerging as prospective precision psychopharmacology tools. Microbiological characteristics can predict SSRI side effects such as gastrointestinal issues and treatment-emergent anxiety (Sjöstedt et al., 2021; Hattori and Yamashiro, 2021). This suggests that microbiome categorization may help doctors identify the appropriate antidepressants, adjust dosages, and improve treatment outcomes using prebiotic and probiotic therapy.

SHIME (Simulator of the Human Intestinal Microbial Ecosystem) shows how SSRIs change gut microbial composition and affect treatment efficacy (Ma et al., 2019; Russo et al., 2018). According to de Oliveira et al. (2025), Mitrea et al. (2022), long-term SSRI exposure can cause dysbiosis, which is characterized by decreased beneficial taxa, metabolic output abnormalities, and increased inflammatory marker levels. Probiotics reduced inflammation, altered metabolic pathways, and restored microbial balance (Mohajeri et al., 2018; Solas et al., 2017). Targeted microbiome modification as an adjuvant method to boost efficacy and decrease side effects gives antidepressant development fresh confidence (Romanescu et al., 2022).

Certain microbial groups have been identified to be associated with neuroinflammation and unresponsiveness to drugs. To illustrate this, the Bacteroides and Clostridium species are linked to a proinflammatory response, whereas Lactobacillus and Bifidobacterium have been linked with anti-inflammatory effects and regulation of neurotransmitters. Low abundance of short-chain fatty acid-producing bacteria like Faecalibacterium prausnitzii has been associated with low response to antidepressants and high neuroinflammation.

### 3.4 Effects of psychotropic drugs on gut microbiome

Mood stabilizers and other psychotropic medicines affect the digestive tract microbiome and CNS (Mejía-Granados et al., 2022; Ma et al., 2020). The study found that amitriptyline and fluoxetine change gut microbial composition, abundance, and metabolism. Both drugs promote proinflammatory bacteria and impair beneficial bacteria like Lactobacillus and Bifidobacterium (Zhang et al., 2021; Dash et al., 2022; Sharon et al., 2016). Antidepressants may affect the microbiota and metabolism systemically due to microbial aberrations that change bile acid, amino acid, and short-chain fatty acid metabolism.

Fluoxetine during pregnancy and nursing changes the mother and child microbiomes (Ramsteijn et al., 2020; Laue et al., 2022). This changes microbiome diversity and metabolomics. These findings highlight the importance of monitoring psychotropic use, especially during key developmental periods. It suggests microbial composition changes affect antidepressant efficacy and acceptability, not simply adverse effects. According to Skonieczna-Żydecka et al. (2018), dysbiosis from medicine can diminish neurotransmitter precursors, hinder absorption, and activate immunological pathways, negating antidepressants’ benefits.

Psychotropic medicines affect CNS drug function through the digestive tract microbiota (Evrensel et al., 2019; Yang et al., 2021). Feedback loops may cause gastrointestinal difficulties, treatment-resistant depression, and tachyphylaxis. Novel adjunctive medications that regulate microbial ecosystems during psychiatric therapy, precision dosage techniques, and microbiome-informed drug development can result from understanding these interactions.

### 3.5 Cannabinoids and microbiome–brain interactions

Cannabinoids, another CNS-active drug, are affected by microbiota. In HIV/SIV rats, microbiome-mediated cannabis treatment improves neurocognition (Fung, 2020). Cannabis lowers intestinal permeability, enhances CNS homeostasis, and reduces systemic inflammation (McDew-White et al., 2023). Greater levels of helpful metabolites like indole-3-propionate and endocannabinoid intermediates complicated the microbiome-endocannabinoid system interaction (Zhu et al., 2020).

Gut microbiome health appears to affect marijuana’s neuroprotective effects on inflammation, oxidative stress, and brain neuronal survival (Dinan and Cryan, 2017; Patrono et al., 2021) as shown in Table 1. These findings show that CNS drugs work within a complex biological framework of microbial populations, immune responses, and metabolic networks. As cannabinoid-based drugs become more common, understanding microbiome-dependent mechanisms may help guide pharmaceutical selection, dosing, and the development of more microbiome-targeted therapeutics (Moya-Pérez et al., 2017).

**TABLE 1:** Microbiome effects on major classes of CNS drugs.

Drug/Class	Microbial metabolism effects	Mechanisms	Impact on efficacy
SSRIs (fluoxetine, sertraline, citalopram)	Bacterial degradation and demethylation; altered absorption	Enzymatic modification; serotonin-modulating gut microbiota; SCFA effects on BBB.	Reduced or variable antidepressant response; improved with psychobiotics
TCAs (amitriptyline)	Microbial conversion to active metabolites; altered recycling	β-glucuronidase activity; microbial metabolites influencing CYPs	Unpredictable plasma levels; increased side effects with dysbiosis
Cannabinoids (THC, CBD)	Microbial alteration of degradation and receptor signaling	Modulation of endocannabinoid pathways and immune signaling	Variable psychotropic and anxiolytic effects
Second-generation antipsychotics (e.g., olanzapine, risperidone)	Microbial metabolism influencing weight-gain pathways	SCFA and bile-acid modulation; metabolic pathway shifts	Altered drug response and metabolic side-effects
Anti-inflammatory neuroprotectants	Enhanced drug stability and uptake mediated by microbial metabolites	Cytokine modulation; oxidative stress reduction; regulation of microglial activation and neuroinflammation	Improved neuroprotection; variable outcomes in dysbiosis

## 4 Microbiome and neuropsychiatric disorders: implications for drug response

Importantly, these microbiome-drug interactions have clinical implications, especially in neuropsychiatric disorders, where changed microbial composition underlies heterogeneity in both disease progression and treatment response.

### 4.1 Depression and stress disorders

Gut-brain axis theory has strengthened empirical evidence relating the gut microbiome to mood disorders. Młynarska et al. (2022), Marazziti et al. (2021) found that gut microbiota composition affects neuron communication, neurotransmitter synthesis, and HPA axis control. All of these contribute to major depressive illness. Dysbiosis activates stress pathways and impairs mood-stabilizing serotonergic and glutamatergic systems, resulting in the common co-occurrence of stress-related disorders frequently co-occur with gastrointestinal disturbances (Delaney and Hornig, 2018; Noble et al., 2017). A rising body of evidence shows that microbial imbalances increase pro-inflammatory cytokines, affect energy metabolism, and impair neuroplasticity in the immune-metabolic axis, which are linked to depression and anxiety.

Dysbiosis has been linked to antidepressant non-response, and particular gut microbial patterns distinguish treatment-resistant individuals from responders. Due to a connection between fewer taxa engaged in SCFA synthesis and tryptophan metabolism and poor SSRI results, microbiome metabolites may affect antidepressant pharmacodynamics. This perspective sees depression as a microbiota, inflammation, and metabolic resilience issue rather than a neurotransmitter imbalance.

#### 4.1.1 Post-traumatic stress disorder (PTSD)

PTSD is increasingly linked to dysfunctional gut microbiome and aberrant stress-response pathways. Emerging evidence indicates that people with PTSD have reduced microbial diversity and changes in relative abundance of key taxa associated with short-chain fatty acid production and immune regulation (Ahmed et al., 2022).

At the mechanistic level, dysbiosis is linked to Hypothalamic-Pituitary-Adrenal (HPA) axis hyperactivation, increased neuroinflammation as well as impaired fear extinction. These shifts affect neural circuits associated with memory and emotional regulation.

From a pharmacological perspective, microbiome changes can impact the effectiveness of widely prescribed treatments like SSRIs by modulating tryptophan metabolism and serotonergic signaling pathways (Jiang et al., 2024). Thus, therapeutics have emerged as microbiome-targeted interventions, probiotics and dietary modulation that could promote treatment response or alleviate disease symptoms. However, longitudinal and mechanistic studies will be required to establish causality and issue clinical relevance.

### 4.2 Autism spectrum disorder (ASD)

One of the most evident gut-brain-microbiota interactions is autism-related gastrointestinal dysfunction and altered microbial profiles. Research suggests that microbial dysbiosis may affect ASD symptoms like anxiety, sensory abnormalities, and social impairment (Zhu et al., 2022). Neuromodulator synthesis, intestinal barrier failure, and immune system dysregulation characterize this condition. Risperidone with SSRIs alleviate ASD irritability, anxiety, and hyperactivity. These drugs may also influence microbial populations, affecting therapy efficacy and safety. The gut’s importance in autism spectrum disorder has led to microbiome-focused therapy (Evrensel and Ceylan, 2015). Long-term follow-ups show that Fecal Microbiota Transplantation (FMT) improves behavioral outcomes, gastrointestinal symptoms, and microbiological and neurological health. Psychobiotic probiotics affect the CNS, improving sleep, social functioning, and anxiety-like behavior (Severance and Yolken, 2020). Available evidence suggests that integrative microbiome management therapy may be effective for patients with poor therapeutic response or unacceptable side effects.

### 4.3 Neurodegenerative diseases

The gut microbiome is increasingly implicated in neurodegenerative diseases like AD (Alzheimer’s disease) and PD (Rutsch et al., 2020; Long-Smith et al., 2020). By increasing neurotoxic metabolite synthesis, decreasing SCFA availability, and increasing systemic inflammation, dysbiosis accelerates amyloid deposition and cognitive loss in AD. In PD, pro-inflammatory taxa-rich microbial profiles may cause α-synuclein misfolding and trigger neuroinflammatory cascades from the stomach to the central nervous system via the vagus nerve (Singh et al., 2022; Liang et al., 2018). These processes support the current notion that neurodegeneration begins in the intestines before symptoms appear.

Microbial metabolites such as H_2_S, glutamate modulators, and dysregulated tryptophan derivatives worsen neurotoxicity and mitochondrial dysfunction (Chakrabarti et al., 2022; Simionescu et al., 2020). Research into microbiome-harmonizing medicines has increased. Nanomedicine methods are the most promising and one example is customized nanoparticles that deliver antioxidants or anti-inflammatory drugs to microbiota-CNS interfaces (James et al., 2021; Bonnechère et al., 2022). These technologies enable medication delivery across blood-brain and gut epithelial barriers with fewer side effects and precise inflammatory pathway modulation (Olayemi, et al., 2025). These advances indicate a new era in neurodegenerative disease treatment that will combine microbiome-focused methods with known drugs.

### 4.4 Acute CNS injury

Gut microbiota affects acute neurological illnesses such as stroke, TBI, and SCI (Gwak and Chang, 2021). Damage to the microbiome swiftly affects it, reducing helpful bacteria and increasing dangerous ones. This produces body-wide inflammation and neurological system damage (Mohammad et al., 2025). Restoring the microbiome boosts neuronal repair and anti-inflammatory immune characteristics. In stroke models, dysbiosis impacts neurogenesis, angiogenesis, and infarct size. In traumatic brain and spinal cord injuries, microbiological metabolites alter neuroimmune communication, glial activation, and synaptic remodeling during recovery.

Interactions between microbiota and CNS repair pathways alter acute injury treatment response. Neurological outcomes following damage can be affected by antibiotics that kill SCFA-producing taxa (Leprun and Clarke, 2019). Microbiome-targeted diets with probiotics improve motor and cognitive rehabilitation. Neurorehabilitation targets the microbiota because it changes and affects injury progression and therapy efficacy.

## 5 Therapeutic modulation of the gut–brain axis in pharmacology

### 5.1 Probiotics, prebiotics, and synbiotics

The gut–brain axis is increasingly regulated therapeutically with probiotics, prebiotics, and synbiotics, which affect CNS function via distinct molecular pathways. By modulating neurotransmitters, immunological signaling, and HPA axis activity, probiotics, especially Lactobacillus and Bifidobacterium species, may alleviate anxiety and depression (Wadan et al., 2025; Wijdeveld et al., 2020). Good bacteria that produce short-chain fatty acids thrive on inulin and fructo-oligosaccharides. Neuron protection and inflammation reduction improve mental and emotional health (Emge et al., 2016). By combining the two techniques, synbiotics boost metabolism, intestinal barrier integrity, and microbial resilience. Psychobiotics often have antidepressant effects these including stress-induced dysbiosis regulation, tryptophan pathway modification, and GABA signaling enhancement (Luna and Foster, 2015). Microbiome-targeting adjuncts may improve mood and stress medicines, notably in dysbiosis patients.

### 5.2 Fecal Microbiota Transplantation (FMT)

FMT is a novel microbiome-based treatment that may enhance neuropsychiatric outcomes by restoring healthy microbial ecosystems (Osadchiy et al., 2019; Talbott et al., 2020). Preclinical and clinical research suggest FMT may improve ASD behavioral and gastrointestinal symptoms, depression, mood modulation, and stress resilience (Nohesara et al., 2025). The metabolically active bacteria that generate SCFAs, modulate immune responses, and replenish neurotransmitter precursors may make FMT effective (Zhang et al., 2025). Despite its potential, FMT has considerable limitations. Due to donor heterogeneity, strain specificity, and engraftment variables, results vary substantially. Safety concerns regarding pathogen transfer and long-term dysbiosis prohibit its widespread usage in mental health treatment (Martin et al., 2018; Hawkins et al., 2020). Thus, scientists are investigating targeted bacteriotherapy and rationally created microbial consortia to regulate gut-brain interactions more precisely.

### 5.3 Diet and nutritional pharmacology

Dietary manipulation is one of the easiest and most effective ways to alter gut flora and central nervous system medications. Ahmed et al. (2022), Hulme et al. (2020) found that fiber, polyphenols, and fermented meals increase SCFAs, indoles, and other neuroactive metabolites. These metabolites affect inflammation, neurotransmitter production, and drug metabolism. Polyphenols can be biotransformed into brain-healthy anti-inflammatory and antioxidant metabolites by microbial metabolism (Mou et al., 2022). In addition, the Mediterranean diet and comparable eating patterns may improve antidepressant response due to metabolic stability and microbial diversity. Nutritional pharmacology helps doctors increase treatment efficacy and reduce side effects by providing individualized meal guidance that strengthens the microbiota and metabolic function (Ejtahed and Hasani-Ranjbar, 2019). Integrative techniques are promising for illnesses with wide therapy variations.

### 5.4 Precision and nanomedicine approaches

Precision medicine is projected to change microbiome-based pharmacology by tailoring CNS treatment regimens using genetic, metabolomic, and microbial data (Mudaliar et al., 2024; Zhang et al., 2018). Individual microbiome profiles are employed as biomarkers to predict medication response, toxicity risk, and treatment resistance for more customized treatments (Krsek et al., 2025; Zainal Abidin et al., 2025). This project has benefited from algorithmic modeling and machine learning. AI-driven prediction systems can currently identify microbiome-mediated illness clusters and predict drug-microbiome interactions (Yuan et al., 2021; Giovannini et al., 2021). Advanced nanomedicine platforms can target microbial metabolites, immunological pathways, or mucosal drug transformation sites, enhancing CNS medication delivery. Nanoparticles that manage dysbiosis or deliver neuroprotective chemicals across the intestinal barrier have improved gut-brain understanding (Kwak et al., 2023). Precision medicine and nanomedicine are transforming microbiome-informed medication, which could improve neurological and psychiatric outcomes.

## 6 Clinical and translational challenges

Although microbiome research has advanced, clinical and translational hurdles limit its integration into CNS pharmacology (Peterson, 2020; Alam, 2025). Because there are no standard microbiological profiling methodologies, sequencing depth, taxonomic resolution, and analytical methods differ between research. This heterogeneity makes patient cohort comparisons and microbiological drug response indicators harder (Matera et al., 2025). Diet, genes, environment, early-life exposures, and comorbidities vary microbiome composition. Diversity makes it hard to draw broad conclusions from studies and develop therapies for all people.

According to Kumar et al. (2024), Tian et al. (2025), Sanli (2024), animal research has illuminated gut-brain communication and drug-microbiome interactions. Changes in microbial richness, immunological function, and neurodevelopmental pathways limit this research’s usefulness. Polypharmacy, nutrition, and chronic disease prevent rat models from replicating human microbiota complexity (Nowacka et al., 2025; Socała et al., 2021). Organoids, SHIME bioreactors, and computer models must be used with *in vivo* discoveries to overcome these restrictions.

One of the methodological weaknesses of microbiome research is that sequencing methods differ. Although 16S rRNA sequencing can be used to give taxonomic information, it is not functional enough relative to shotgun metagenomics, which can be used to analyze each microbial gene and metabolic pathways in detail. Furthermore, the majority of the studies are cross-sectional, which does not allow making causal inferences. Longitudinal design is needed to learn about the changes of the microbiomes with time and pharmaceutical interactions. Other confounding variables like diet, exposure to antibiotics and lifestyle make the interpretation further complicated and have to be given standardized protocols in future clinical studies.

Lack of pharmacokinetics and microbiota clinical trials is another issue; most research analyzes microbiome metrics individually, so it is unknown how inflammatory circumstances, microbial enzymes, and drug metabolism affect CNS medicine absorption, distribution, and clearance (Stopińska et al., 2021; Tremblay et al., 2021). Without methodology, biomarker validation frameworks, and longitudinal microbiome monitoring, microbiome-informed therapy cannot be clinically used. These obstacles require interdisciplinary teams, standardised techniques, and regulatory guidance to translate microbiome research to specific central nervous system therapeutics.

In a clinical context, microbiome profiling might be adopted using stool-based sequencing combined with pharmacogenomic tests guiding CNS drug selection and dosing. For example, probiotic adjunctive therapy combined with antidepressant treatment may be beneficial in patients with reduced co-occurrences of short-chain fatty acids-producing intestinal bacteria.

Need standardized biomarkers, cost-effective diagnostics and regulatory frameworks for implementation. Integration of these approaches into routine clinical practice is contingent upon their validation in large-scale, prospective clinical trials and subsequent development of clinically actionable guidelines.

### 6.1 Sex-based variability in microbiome–drug interactions

New findings suggest that gender disparities in intestinal microbiome structure could profoundly affect the metabolism of drugs and neuropsychiatric effects. The hormonal changes, especially in estrogen and androgens, have been known to control the diversity of microbes and their metabolic rate, thus influencing host-microbiome interactions and variability in drug responses (Dinan and Cryan, 2017).

The effect of hormones on microbial enzyme activity, such as drug metabolism and immune modulation pathways can affect pharmacokinetics and therapy response. Such interactions are especially pertinent in CNS disorders, where microbiome-mediated processes lead to differences in the effects of antidepressants, side-effects, and neuroinflammatory reactions (Sherwin et al., 2016).

Nevertheless, the existing evidence is still sparse, and additional sex-stratified clinical and translational research is needed to provide mechanistic clarity and applicability to clinical practice.

### 6.2 Critical evaluation and limitations of current evidence

Despite rapid advancements, the current evidence is constrained by methodological heterogeneities in microbiome sequencing methodologies, small sample sizes, and the absence of standardized analytical frameworks. Most discoveries come from preclinical animal models that may not accurately reproduce the complexity of the human microbiome (Socała et al., 2021; Peterson, 2020).

There are conflicting results on the impact of microbiota on drug efficacy, either increasing or decreasing it, which is influenced by heterogeneity between study populations, microbiome composition and drug classes (Lee et al., 2022). Most studies are also cross-sectional, making it difficult to determine causal links between variables.

The interplay between host genetics and microbiome community structure has received little attention, making it difficult to interpret pharmacological results. Further studies on high-throughput longitudinal data combining microbiome, metabolomic and pharmacogenomics are needed to achieve higher predictive ability while maintaining clinical relevance.

## 7 Future directions

The next decade of CNS pharmacology will focus on microbiome-informed pharmaceutical design, supported by computational tools, clinical innovation, and mechanistic study. To determine how microbial states affect therapeutic efficacy and toxicity, use longitudinal microbiome sequencing, metabolomics, and pharmacokinetic profiling in future clinical studies. Predictive algorithms for tailored treatment can leverage multidimensional data.

BERT and other Natural Language Processing (NLP) models are increasingly used for literature mining and text-based classification in microbiome research. However, multimodal AI approaches integrating genomic, metabolomic, and clinical data are required for more accurate prediction of microbiome–drug interactions. Targeted therapy is possible by identifying microbial clusters linked to neuroinflammatory vulnerability, treatment-emergent adverse effects, or antidepressant resistance. Engineered probiotics, consortia, and neuron-specific metabolite-delivery systems are being explored alongside next-generation microbial treatments.

Polypharmacy-microbiome interactions are critically needed since neurological and mental illnesses require many regimens. To ensure safe and effective CNS therapy, one must understand how drugs impact microbial ecology and how dysbiosis influences efficacy. These exciting new research methods promise a future in neurology and psychiatry where the microbiome dictates diagnosis, therapy, and prognosis.

## 8 Conclusion

This review outlines how the gut microbiome acts as a complex and important regulator of CNS pharmacology, impacting both the pharmacokinetics and pharmacodynamics of neuropsychiatric drugs. The interaction between microbiota and pharmacological agents is bidirectional, contributing to variability in treatment response and disease outcomes.

Future studies should incorporate long-term clinical follow-up, integration of microbiome and pharmacogenomic data, including validation of microbial biomarkers for clinical application. Microbiome-targeted therapies, including engineered probiotics and precision nutrition, are promising for personalized neuropsychiatric treatment.

Merging microbiome studies into clinical pharmacology, where potential to refine therapeutic strategies, could then directly impact neuropsychiatric disorders.
